# Association of Metabolic Syndrome and Inflammation with Cognitive Decline in Adults Aged 60 Years and Older: Findings from a National Health Survey in the United States

**DOI:** 10.1155/2013/846027

**Published:** 2013-12-26

**Authors:** Zuolu Liu, Carol F. Lippa

**Affiliations:** ^1^Temple University School of Medicine, 2720 Springhouse Road, Philadelphia, PA 19140, USA; ^2^Department of Neurology, Drexel University College of Medicine, Philadelphia, PA 19102, USA

## Abstract

*Objectives*. We aimed to test the hypothesis that metabolic syndrome (MetS) is significantly associated with cognitive decline (CoD) in elderly adults and further assess whether MetS and inflammation have a significant joint effect on CoD. *Methods*. Data (*n* = 2975) from the U.S. National Health and Nutrition Examination Survey (1999–2002) in participants aged ≥60 years who had Digit Symbol Substitution Tests (DSS: a standard measure of cognitive function) were studied. CoD was defined as those in the lowest quintile of DSS score. MetS was defined as having ≥3 of 5 MetS traits (large waist circumference (WC), high blood pressure (BP), elevated glucose, triglycerides, and decreased high density lipoprotein cholesterol). *Results*. Of 2975 participants, the prevalence of CoD (DSS score <25) was 12.1%. After adjusting covariates, individual large WC, high BP, elevated glucose level, and MetS were significantly associated with CoD in logistic regression models (*P* < 0.001). There was a significant dose-response relationship between an increased number of MetS traits and CoD (*P* < 0.001). A significant joint effect of MetS and CRP on the odds of CoD was observed. *Conclusion*. The study, using a nationally representative sample, extended previous studies by highlighting a significant MetS-CoD relationship and a joint effect of MetS and CRP on CoD. These novel findings add to our understanding of the association of neurometabolic disorders and cognition and have implications that may be relevant to primary care practice.

## 1. Introduction

Impaired cognitive function is emerging as one of the greatest health threats of the twenty-first century. As the life expectancy of the population has increased so too has the prevalence of cognitive decline (CoD) and dementia, largely in the form of Alzheimer's disease, which now affects almost 50% of adults over the age of 85 in the United States [1]. This startling figure will only grow as the average age of the population rises, so understanding the basis of CoD during ageing is critical [2]. Metabolic syndrome (MetS), a cluster of cofactors (large wait circumference, high blood pressure, elevated triglycerides, impaired glucose metabolism, and decreased high density lipoprotein cholesterol), and CoD are prevalent concomitants of aging. Cognitive function has been found to predict subsequent disability and mortality in elderly adults [3]. Although potential mechanisms remain obscure, they may include insulin resistance and inflammation leading to atherosclerosis, oxidative DNA damage, and CoD [2, 4]. For example, some studies have shown that insulin/insulin-like growth factor 1 (IGF-1) is neurotrophic and promotes neuronal survival by inhibiting apoptosis. Insulin and IGF-1 can also promote learning and memory in humans and animal models [5, 6]. However, information regarding the associations of MetS and inflammation with cognitive function is scarce in studies using large-scale representative elderly samples. In the present study, we tested the hypothesis that MetS and its individual traits are significantly associated with cognitive decline and that this association is mediated by inflammation. We further assessed whether MetS and inflammation have a significant joint effect on the function of cognition. We analyzed data from the National Health and Nutrition Examination Surveys (NHANES) that are conducted using nationally representative samples and state-of-the-art interviewing, examination, and laboratory methods [7].

## 2. Study Design and Methods

### 2.1. Study Design and Population

The NHANES, using a cross-sectional study design, has been conducted since 1971. This study has been redesigned to conduct and combine data once in a two-year cycle since 1999. In the NHAES, participants are selected using a multiple sampling approach to recruit a nationally representative sample of approximately 10,000 participants aged 2 months and older in each two-year cycle [7]. In the present study, we used data from the 1999-2000 and 2001-2002 NHANES that included cognitive function tests for participants aged 60 and older [7]. Of 3702 participants, 727 elderly adults without cognition tests were excluded, so the final analysis sample size used in the study was 2975 (M: 1438, F: 1537). The NHANES has been approved by the Institute Review Board of the U.S. Centers for Disease Control and Prevention. Data used in the present study is deidentified and publicly available for researchers [7].

The NHANES consists of interviews, physical examinations, and data from blood sample analysis. All measurements were processed per standard protocols. Household interviews were conducted prior to the examination to collect demographic, medical history, and health behavioral information. Race and ethnicity were determined by self reports. Examinations were carried out in mobile examination centers. Blood samples were obtained at the examination centers. Participants were asked to fast for 12 hours prior to the morning examination or 6 hours prior to the afternoon or evening examination. To ensure the quality of blood sample collection and to minimize hemolysis of blood samples, all phlebotomists were certified and trained in standardized laboratory procedures. Serum lipid profiles, metabolic indicators, and CRP concentrations were measured using standard methods. Chronic conditions, including hypertension, coronary heart disease, diabetes mellitus, and stroke were participants' self-reported medical history that were diagnosed by a physician or health professional. A detailed description of the survey and its sampling procedure is available at the NHANES website [7].

### 2.2. Cognition Function Assessment

In the 1999–2002 NHANES, cognitive function was tested using the WAIS III (Wechsler Adult Intelligence Scale, Third Edition) Digit Symbol Substitution Test (DSS)—Coding module and was conducted during the household interview for those aged 60 years and older. In this coding exercise of DSS, participants copy symbols that are paired with numbers. Using the key provided at the top of the exercise form, the participant draws the symbol under the corresponding number. The score is the number of correct symbols drawn within a period of 120 seconds. One point is given for each correctly drawn symbol completed within the time limit. The maximum score is 133 with a lower score indicating poorer cognitive function. Sample items are provided for initial practice. Participants who are unable to complete any of the sample items do not continue with the remainder of the exercise [7].

### 2.3. Metabolic Traits, Definition of Metabolic Syndrome, and C-Reactive Protein

Several criteria for diagnosing MetS have been proposed by health organizations [8, 9]. In the present study, we applied the criteria proposed by the US National Cholesterol Education Program (NCEP) Expert Panel III (2001) with minor modifications from the criteria proposed by the American Heart Association (AHA 2005). The criteria inform the identification of MetS in the presence of ≥3 of the following 5 traits: (a) waist circumference (WC) >102 cm in men or >88 cm in women, (b) fasting plasma glucose ≥110 mg/dL (6.1 mmol/L), (c) systolic/diastolic blood pressure (SBP/DBP) ≥130/85 mmHg, (d) elevated triglycerides (TG) ≥150 mg/dL (1.7 mmol/L), and (e) high density lipoprotein cholesterol (HDL-C) <40 mg/dL (1.0 mmol/L) in men or <50 mg/dL (1.3 mmol/L) in women. In addition, participants who reported current use of antihypertensive or antidiabetic medication (insulin or oral agent) or previous diagnosis of hypertension or diabetes are considered to have evidence of elevated blood pressure or raised fasting glucose and are included in the sample regardless of measured blood pressure or fasting glucose values at the time of the examination. Serum CRP concentration was measured at the Immunology Division of the University of Washington (Seattle, WA) with latex-enhanced nephelometry using a Behring Nephelometer Analyzer System (Behring Diagnostics Inc., Somerville, NJ).

### 2.4. Covariates

In the analysis, we included the following variables as covariatesage (years), sex (male or female), race/ethnicity (White, Black, Hispanic, and others), marital status (married, widowed, separated/divorced, or single), and smoking status (ever-smoking versus never).

### 2.5. Statistics Analysis

A serial analysis was conducted using univariate and multivariate analysis approaches to adjust for potential confounders. In the first group analysis, we described the characteristics of the study participants by sex. Differences in the study variables between genders were tested using *t*-test for continuous variables, and Chi-square for categorical variables. Serum TG, glucose, and CRP concentrations are log-transformed because their values have a skewed distribution. The relationship between age and DSS score is tested using linear regression analysis for White, Black, and Hispanic groups separately. In the second group analysis, we classified participants into 5 groups by quintiles of the distribution of DSS scores. Subjects who had DSS scores in the lowest quintile (i.e., DSS score <25) were classified as having CoD. We then tested adjusted mean differences in each MetS trait and CRP across quintiles of DSS using general linear models, with adjustment for age, sex, and race/ethnicity. Trends of changes in means of MetS traits and CRP across DSS quintiles were tested using linear regression analysis. In the third group analysis, we estimated odds ratios of individual MetS traits and MetS for the prevalence of CoD using multivariate logistic regression modeling technique with adjustment for a set of covariates. Then, three models were conducted using the total combined sample of all racial/ethnic groups: (1) in Model 1, age, sex, and race/ethnicity were adjusted, (2) in Model 2, age, sex, race/ethnicity, marital status, and cigarette smoking status were adjusted, and (3) in Model 3, the covariates that were adjusted in Model 2 plus CRP were adjusted in order to test any potential mediate effect of CRP with MetS on CoD. In this analysis, serum CRP level was dichotomized. Subjects who had serum CRP values in or higher than 75% distribution in the total study participants were classified as having a high CRP value (>0.60 mg/L). In the final analysis, we examined the combined effects of MetS (yes versus no) and CRP (>0.60 mg/dL versus ≤0.60 mg/L) on CoD by three ages (60–69, 70–79, and ≥80) and sex.

All data analyses were conducted using SAS software version 9.2 (SAS Institute, Cary, NC). SAS Statistics package for Surveymean, Surveyfreq, Surveyreg, and Surveylogistic procedures were used to take into consideration the complex multistage sampling data collection used in the NHANES. A two-sided *P* value <0.05 was considered as having statistical significance.

## 3. Results

Table 1 shows that there were significant differences in age, individual MetS traits, CRP, race/ethnicity, marital status, smoking status, and chronic conditions between men and women (*P* < 0.05, or <0.01), except for the differences in serum TG and the prevalence of stroke and diabetes (*P* > 0.05).

**Figure 1:**
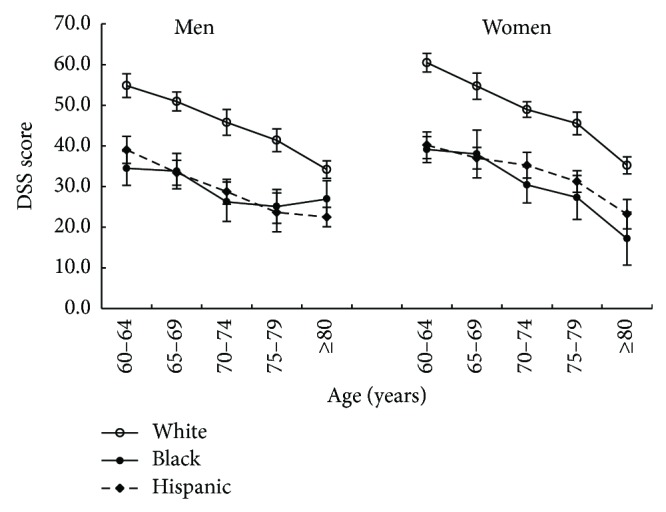
Mean DSS score by age, sex, and race/ethnicity.

Of 2975 participants aged ≥60, the prevalence of CoD (DSS score <25) was 12.4% in males, and 11.79% in females (*P* = 0.02).  Figure 1 depicts a significant decreased trend of mean DSS scores from younger to older age groups (test for trend, *P* < 0.001). Black and Hispanic had significantly lower mean DSS scores than White across age groups (*P* < 0.01).

Table 2 shows that age, sex, and race-adjusted means of individual MetS traits and CRP significantly increased from the lowest to the highest quantile of DSS scores (*P* < 0.01), except for nonsignificant changes in mean DBP (*P* = 0.158) and log-TG values (*P* = 0.144).

Table 3 shows that 3 of 5 MetS traits (large WC, high BP, and elevated glucose) had significantly higher odds ratios for CoD than their corresponding comparison groups (Models 1–3). After adjustment for CRP, the values of odds ratios for each individual MetS trait decreased slightly (Model 3). However, adjusting CRP resulted in an increased odds ratio of MetS for CoD from 1.40 (1.03–1.90) in Model 2 to 1.68 (1.25–2.24) in Model 3. A dose-response association between an increased number (1 to ≥4) of unfavorable MetS traits and odds ratios for CoD was observed (test for trend, *P* < 0.001, Models 1–3). The values of odds ratios in those with ≥3 MetS traits combination significantly increased after adjustment including CRP.

**Figure 2:**
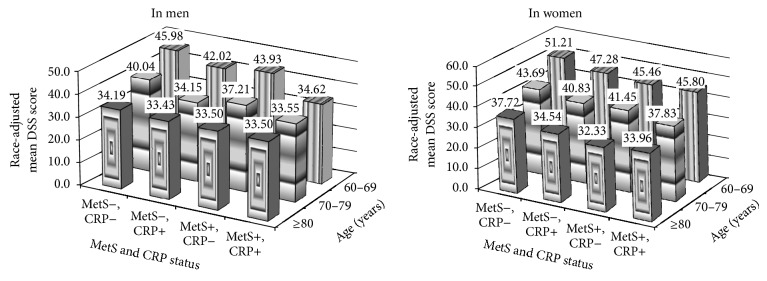
Joint effects of metabolic syndrome (MetS) and C-reactive protein (CRP) on cognitive decline by age in men and women.

Figure 2 depicts a significant combined effect of MetS and CRP on CoD across subjects aged 60–69, 70–79, and ≥80 years old in men and women. The joint effects were different in men and in women. For example, males who had MetS and elevated CRP had approximately lower DSS scores regardless of aging (34.6, 33.6, and 33.5 in those aged 60–69, 70–79, and ≥80, resp.), but these scores significantly varied in women (45.8, 37.8, and 34.0 in those aged 60–69, 70–79, and ≥80, resp.).

## 4. Discussion

The main findings from the study are that MetS significantly predicts the odds for the prevalence of CoD in subjects aged ≥60 years old. Of 5 MetS traits, large WC (a marker of central obesity), high BP, and impaired glucose metabolism had significantly independent effects on the risk of the prevalence of CoD. Elevated CRP had potential meditative and combined effects with MetS on the risk of CoD. These associations were independent of a set of covariates. These findings add to growing evidence that aggressively controlling MetS and CRP may offer an important primary prevention strategy to prevent or attenuate CoD in the elderly.

MetS, first described about 40 years ago, is a multifactorial disorder of substantial heterogeneity, represented by a concurrence to central obesity that also includes impaired glucose metabolism, dyslipidemia, and high BP and that depicts a risk status for both type 2 diabetes mellitus, and coronary artery disease [10]. A possible role of MetS was recently proposed for age-related changes of cognitive function and predementia syndromes [11–13]. Results from the present analysis are consistent with most studies that observed a significantly positive association between MetS and CoD, [4, 11, 14] but inconsistent with a nonsignificant association report from the European Male Ageing Study by Dr. Tournoy and colleagues [15]. The possible explanation of this nonsignificant association by Tournoy et al. might be due to potential selection bias of participants and possible limitations in their statistical data analysis. For example, the overall response rate of the European Male Ageing Study was 41%. It is significantly lower compared to the corresponding rate of more than 65% in the U.S. 1999–2002 NHANES. In their analysis, depression was adjusted in multivariate models, which might lead to an over adjustment because depression is likely on the etiological pathway of MetS → depression → CoD, or there is a reversed association between CoD and depression [16, 17].

MetS and 3 of 5 MetS traits, central obesity, high BP, and elevated fasting glucose had significant associations with CoD in our present study. There have been several reports of MetS link to deficits in memory, executive functioning, processing speed, and overall intellectual functioning, although the potential mechanisms by which MetS and its traits are associated with the risk of CoD still need to be studied using data from large-scale prospective studies and clinical trials [11–14, 18].

To our knowledge, the present study is the first to test a dose-response association between an increased number of MetS traits and CoD using a nationally representative sample in elderly adults aged 60 and older. This finding highlights the magnitude of the number of MetS traits on CoD. Furthermore, a significant joint effect of MetS and CRP on CoD is observed. The degrees of this joint effect were different in men and women. It appeared that it had a similar effect in those with MetS and elevated CRP in men regardless ageing, but it varied in women. Although the present study is unable to test the mechanism of the associations of MetS and CRP with CoD, the findings of the study provide us with important evidence that aggressive treatment for MetS and elevated CRP could have immediate protect effects on improving cognitive function.

Several limitations may exist in the present study. Firstly, this study used data from baseline 1999–2002 NHANES sample that had a cross-sectional study design. Therefore, it cannot be applied to interpret any causal-effect relationship of MetS and CRP with CoD. Secondly, although a significant difference in mean DSS scores was observed by racial/ethnic groups (Figure 1), we are unable to test the details by race/ethnicity, because the subsamples sizes of Black (*n* = 428) and Hispanic (*n* = 557) were much smaller than White (*n* = 1823). Further studies are needed to address in-depth racial/ethnic differences in MetS and cognitive function. Finally, the associations between MetS, CRP, and CoD may be underestimated due to possible selection and survival biases. For example, subjects who had serious disease status, including those who were hospitalized or died, were not included in the NHANES.

There are also several advantages of the present study. Firstly, regarding studies in elderly populations, the present study is one of the few large-scale studies that had a nationally representative sample of those aged 60 years and older. Secondly, the study takes the advantages of the NHANES design, data collection, measurements, and lab tests that are validated and standardized under the support of the National Center for Disease Control and Prevention. Finally, using a standard and robust statistics analysis approach, the present study has demonstrated a strong and significant dose-response relationship between MetS traits and CoD that enhances the prevention of CoD through treatment of MetS.

In conclusion, the study, using a nationally representative sample, extended previous studies by highlighting a significant MetS-CoD relationship and a joint effect of MetS and CRP on CoD. These novel findings add to our understanding of the association of neurometabolic disorders and cognition and have implications that may be relevant to primary care practice.

## Figures and Tables

**Table 1:** Characteristics of participants.

	Men		Women		
	(*n* = 1438)		(*n* = 1537)		*P* value
	Mean/%	(SEM/P)	Mean/%	(SEM/P)	
Continuous var. mean (SEM)					
Age (yrs)	70.04	(0.26)	71.17	(0.37)	0.002
Waist circumference (mm)	104.00	(0.36)	96.21	(0.42)	<0.001
Systolic BP (mmHg)	133.90	(0.70)	141.94	(0.97)	<0.001
Diastolic BP (mmHg)	71.61	(0.50)	69.87	(0.55)	0.015
HDL-C (mg/dL)	46.50	(0.60)	58.95	(0.61)	<0.001
Triglycerides (mg/dL)	150.96	(3.40)	150.98	(3.09)	0.264
Fasting glucose (mg/dL)	106.09	(1.25)	101.53	(1.06)	0.003
C-reactive protein (mg/L)	0.50	(0.04)	0.55	(0.02)	<0.001
DSS test score	45.26	(0.78)	47.03	(0.78)	0.026
Categorical var. (%) (SEP)					
Race/ethnicity					<0.001
White	84.77	(1.66)	82.80	(2.07)	
Black	6.35	(0.91)	7.12	(1.29)	
Hispanic	2.89	(0.54)	2.63	(0.61)	
Others	6.00	(1.39)	7.45	(1.78)	
Marital status					<0.001
Married	78.89	(1.54)	46.52	(1.81)	
Unmarried	21.11	(1.54)	53.48	(1.81)	
Smoking status					<0.001
Never smoked	30.41	(1.72)	60.50	(1.86)	
Formal smokers	57.49	(1.49)	30.30	(1.65)	
Current smokers	12.10	(0.98)	9.20	(0.91)	
Chronic conditions					
Hypertension	30.81	(1.36)	44.56	(1.79)	<0.001
Coronary heart disease	13.83	(1.26)	7.05	(1.01)	0.001
Stroke	5.93	(0.67)	6.17	(0.62)	0.798
Diabetes	14.82	(1.26)	13.43	(0.86)	0.432
MetS (%)	34.24	(1.48)	39.78	(1.77)	0.019

SEM/P: standard error of mean, standard error of proportion. HDL-C: high density lipoprotein cholesterol.

DSS: digit symbol substitution test. Sex differences were tested using SAS procedure Surveyreg.

Log-triglyceride, log-glucose, and log-C-reactive protein values were used in the test.

MetS: metabolic syndrome.

**Table 2:** Adjusted means of individual MetS components and inflammatory markers across quintiles of DSST scores.

	DSST score by quintiles (*N*: range of DSS score)										
	Q1		Q2		Q3		Q4		Q5		Trend test, adjusted *P* value
	(575 : 58–117)		(600 : 46–57)		(641 : 35–45)		(556 : 25–34)		(603 : 0–24)	
	Mean	(SE)	Mean	(SE)	Mean	(SE)	Mean	(SE)	Mean	(SE)
MetS components											
WC (mm)	97.57	(0.60)	100.43	(0.56)	100.09	(0.54)	99.97	(0.61)	101.04	(0.62)	<0.001
SBP (mmHg)	137.09	(1.00)	138.82	(0.93)	138.56	(0.91)	142.60	(1.01)	143.02	(1.02)	0.003
DBP (mmHg)	71.34	(0.57)	70.74	(0.53)	69.11	(0.52)	70.23	(0.58)	70.64	(0.59)	0.158
HDL-C (mg/dL)	54.47	(0.72)	52.32	(0.67)	52.40	(0.65)	51.19	(0.72)	51.61	(0.75)	0.002
Log-TG (mg/dL)	4.87	(0.02)	4.88	(0.02)	4.91	(0.02)	4.86	(0.02)	4.88	(0.02)	0.144
Log-glucose (md/dL)	4.58	(0.01)	4.60	(0.01)	4.63	(0.01)	4.66	(0.01)	4.65	(0.01)	<0.001
Inflammatory markers											
Log-CRP (mg/L)	0.32	(0.02)	0.35	(0.02)	0.35	(0.01)	0.39	(0.02)	0.41	(0.02)	0.001

MetS: metabolic syndrome; DSST: digit symbol substitution test. Q1 to Q5: the first to fifth quintiles of DSS scores.

WC: waist circumference; SBP: systolic BP; DBP: diastolic BP; HDL: high density lipoprotein cholesterol; TG: triglycerides.

Trend test for age, sex, and race/ethnicity-adjusted.

**Table 3:** Odds ratios (95% CI) of individual MetS traits and the number of MetS for cognitive decline.

	Model 1			Model 2			Model 3		
	OR	(95% CI)	*P* value	OR	(95% CI)	*P* value	OR	(95% CI)	*P* value
Individual MetS traits									
Large WC versus small	1.54	(1.23–1.93)	<0.001	1.52	(1.22–1.90)	<0.001	1.52	(1.18–1.95)	0.001
High BP versus low	1.47	(1.14–1.90)	0.003	1.49	(1.15–1.92)	0.003	1.42	(1.05–1.92)	0.024
Decreased HDL-C versus high	1.27	(0.91–1.77)	0.154	1.27	(0.90–1.77)	0.170	1.23	(0.88–1.73)	0.233
Elevated TG versus low	1.24	(0.94–1.64)	0.125	1.25	(0.96–1.64)	0.104	1.23	(0.94–1.61)	0.134
Elevated glucose versus low	1.94	(1.40–2.68)	<0.001	1.87	(1.36–2.57)	<0.001	1.76	(1.23–2.52)	0.002
MetS: Yes versus No	1.41	(1.03–1.92)	0.032	1.40	(1.03–1.90)	0.034	1.68	(1.25–2.24)	0.001
No. of MetS traits									
1 versus 0	1.29	(0.78–2.13)	0.328	1.32	(0.79–2.22)	0.294	1.24	(0.60–2.55)	0.568
2 versus 0	1.35	(0.84–2.17)	0.219	1.35	(0.83–2.19)	0.226	1.38	(0.71–2.67)	0.339
3 versus 0	1.72	(1.04–2.85)	0.036	1.72	(1.05–2.81)	0.031	2.02	(1.09–3.75)	0.026
≥4 versus 0	1.88	(1.17–3.02)	0.009	1.90	(1.18–3.06)	0.008	2.25	(1.22–4.16)	0.010
Test for trend	1.16	(1.04–1.29)	0.005	1.16	(1.04–1.28)	0.006	1.24	(1.12–1.38)	<0.001

MetS: metabolic syndrome; WC: waist circumference; BP: blood pressure; HDL-C: high density lipoprotein cholesterol; TG: triglyceride.

Model 1: adjusted for age, sex, and race.

Model 2: adjusted for covariates in Model 1 plus marital status and smoking status.

Model 3: adjusted for covariates in Model 2 plus log-CRP.
